# Supplementary material for: Non-glycosylated IGF2 prohormones are more mitogenic than native IGF2
Source: Commun Biol. 2023 Aug 19;6:863. doi: 10.1038/s42003-023-05239-6 (PMC10439913; doi:10.1038/s42003-023-05239-6)
Supplement: Supplementary file 2 — Supplementary Information [file 42003_2023_5239_MOESM2_ESM.pdf]

Non-glycosylated IGF2 prohormones are more mitogenic than native IGF2

Pavlo Potalitsyn[1,2], Lucie Mrázková[1,3], Irena Selicharová[1], Michaela Tencerová[4], Michaela Ferenčáková[4], Martina Chrudinová[1], Tereza Turnovská[1], Andrzej Marek Brzozowski[5], Aleš Marek[1], Jakub Kaminský[1], Jiří Jiráček[1*], Lenka Žáková[1*]

[1]Institute of Organic Chemistry and Biochemistry, Czech Academy of Sciences, Flemingovo nám. 2, 116 10 Prague 6, Czech Republic

Departments of [2]Biochemistry and [3]Cell Biology, Faculty of Science, Charles University, 12840, Prague 2, Czech Republic

[4]Institute of Physiology, Czech Academy of Sciences, Vídeňská 1083, Prague 4, Czech Republic

[5]York Structural Biology Laboratory, Department of Chemistry, University of York, Heslington, York YO10 5DD, United Kingdom

Contents

Supplementary Fig. S1 RP-HPLC chromatograms of purified IGF2 proforms 3

Supplementary Fig. S2 MS spectra of purified IGF2 proforms 4

Supplementary Note 1 Determination of secondary structures of IGF2 and IGF2 prohormones by Circular Dichroism and theoretical calculations 6

Supplementary Table S1 Secondary structure content (in %) 8

Supplementary Fig. S3 Comparison of IGF2 NMR structure with predicted structures of IGF2 and big-IGF2(87) 9

Supplementary Fig. S4 Analysis of predicted IGF2 structure 9

Supplementary Fig. S5 Analysis of structures predicted using AlphaFold2 10

Supplementary Table S2 Secondary structure content (in %) 11

Supplementary Table S3 Receptor-binding affinities of IGF2 proforms 12

Supplementary Fig. S6 Binding curves of IGF2 proforms for IR-A, IR-B, IGF1-R and R- 13

Supplementary Fig. S7 Saturation binding curves of [^{125}I]-monoiodotyrosyl-Tyr2-IGF2 and [^{125}I]-monoiodotyrosyl-TyrA14-insulin on R-cells 14

Supplementary Fig. S8 Saturation binding curve of [^{125}I]-monoiodotyrosyl-Tyr2-IGF2 on immobilized IGFBP3 14

Supplementary Fig. S9 Saturation binding curve of [^{125}I]-monoiodotyrosyl-Tyr2-IGF2 on immobilized IGFBP3:ALS complex 15

Supplementary Fig. S10 Binding curves of IGF2 proforms on D11:IGF2R, IGFBP3, ALS:IGFBP3 16

Supplementary Fig. S11 Binding curves of glycosylated and non-glycosylated pro-IGF2(156) on IR-A, IGF1-R, IGF2R on IGFBP3 17

Supplementary Fig. S12 Representative and uncropped Western blots for relative abilities of IGF2 proforms to stimulate receptors' phosphorylation 18

Supplementary Fig. S13 Representative and uncropped Western blots showing expression of particular receptors in different cells. 23

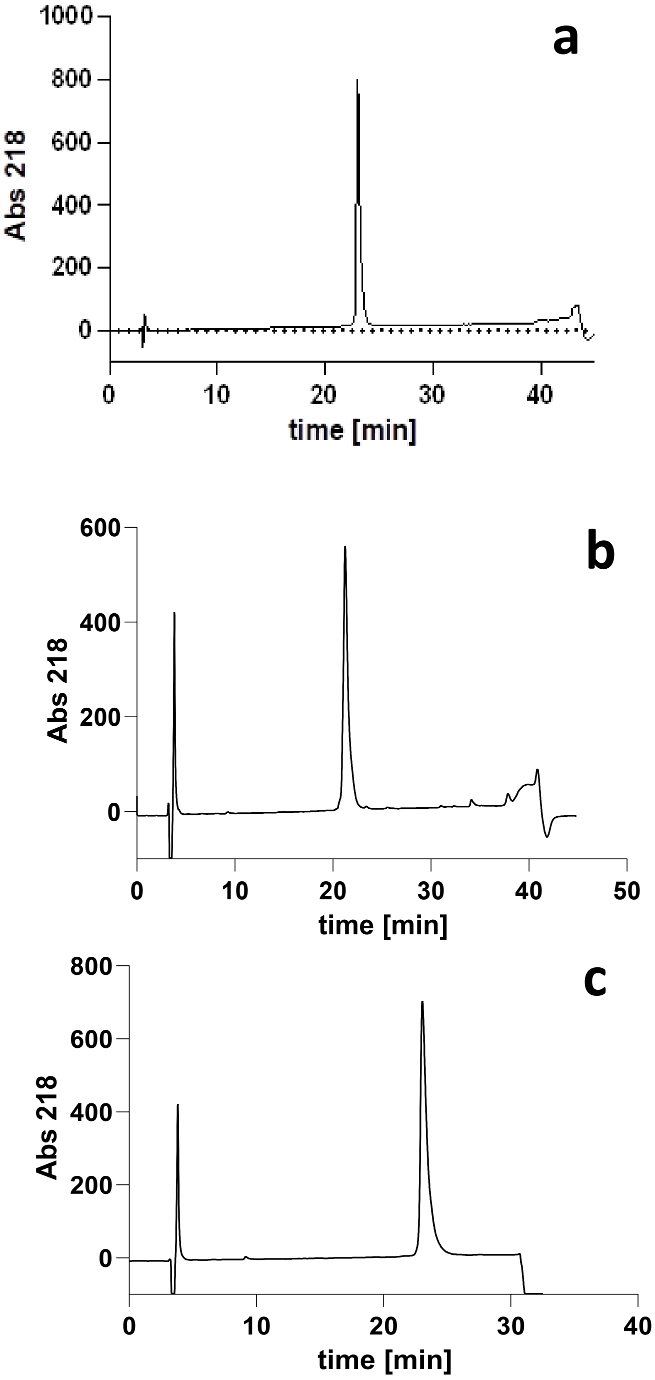

Supplementary Fig. S1 RP-HPLC chromatogram of the purified (**a**) big-IGF2(87), (**b**) big-IGF2(104), and (**c**) pro-IGF2(156). Analysis was done on Vydac C4 column 0.4 x 25 cm in a gradient of acetonitrile in water with 0.1 % TFA (Solvent A: H2O with 0.1% TFA; Solvent B: 80% acetonitrile in H2O with 0.1% TFA. 0 min/25% B, 33 min/55% B, 34 min/100% B, 36 min/100% B, 37 min/25% B).

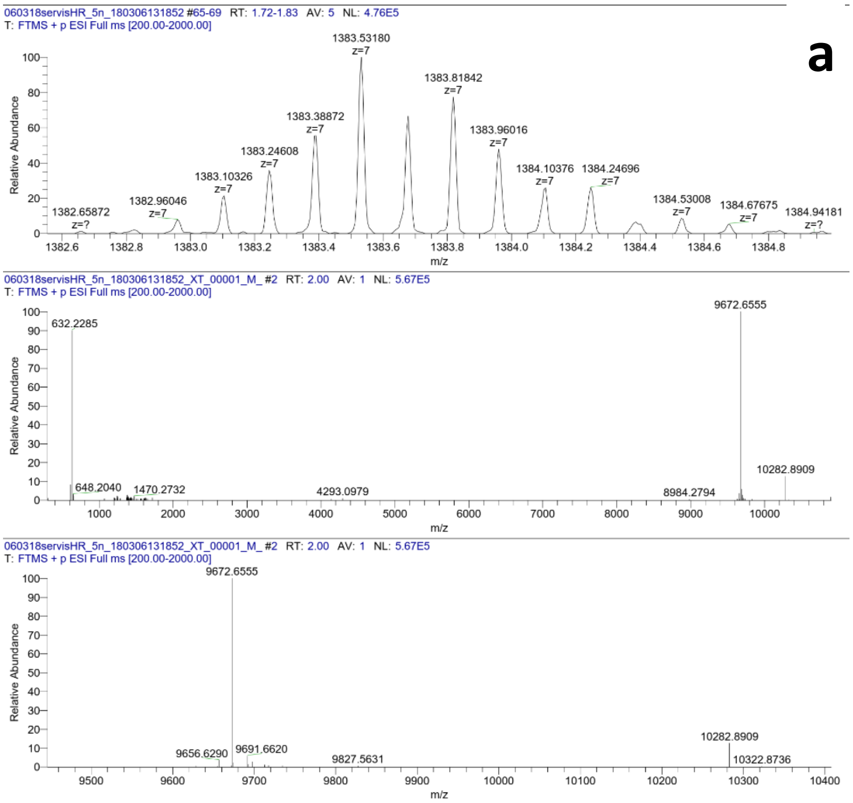

a
060318servisHR_5n_180306131852 #65-69 RT: 1.72-1.83 AV: 5 NL: 4.76E5
T: FTMS + p ESI Full ms [200.00-2000.00]
Relative Abundance
1382.65872 z=?
1382.96046 z=7
1383.10326 z=7
1383.24608 z=7
1383.38872 z=7
1383.53180 z=7
1383.81842 z=7
1383.96016 z=7
1384.10376 z=7
1384.24696 z=7
1384.53008 z=7
1384.67675 z=7
1384.94181 z=?
m/z
060318servisHR_5n_180306131852_XT_00001_M_#2 RT: 2.00 AV: 1 NL: 5.67E5
T: FTMS + p ESI Full ms [200.00-2000.00]
632.2285
648.2040
1470.2732
4293.0979
8984.2794
9672.6555
10282.8909
m/z
060318servisHR_5n_180306131852_XT_00001_M_#2 RT: 2.00 AV: 1 NL: 5.67E5
T: FTMS + p ESI Full ms [200.00-2000.00]
9672.6555
9656.6290
9691.6620
9827.5631
10282.8909
10322.8736
m/z

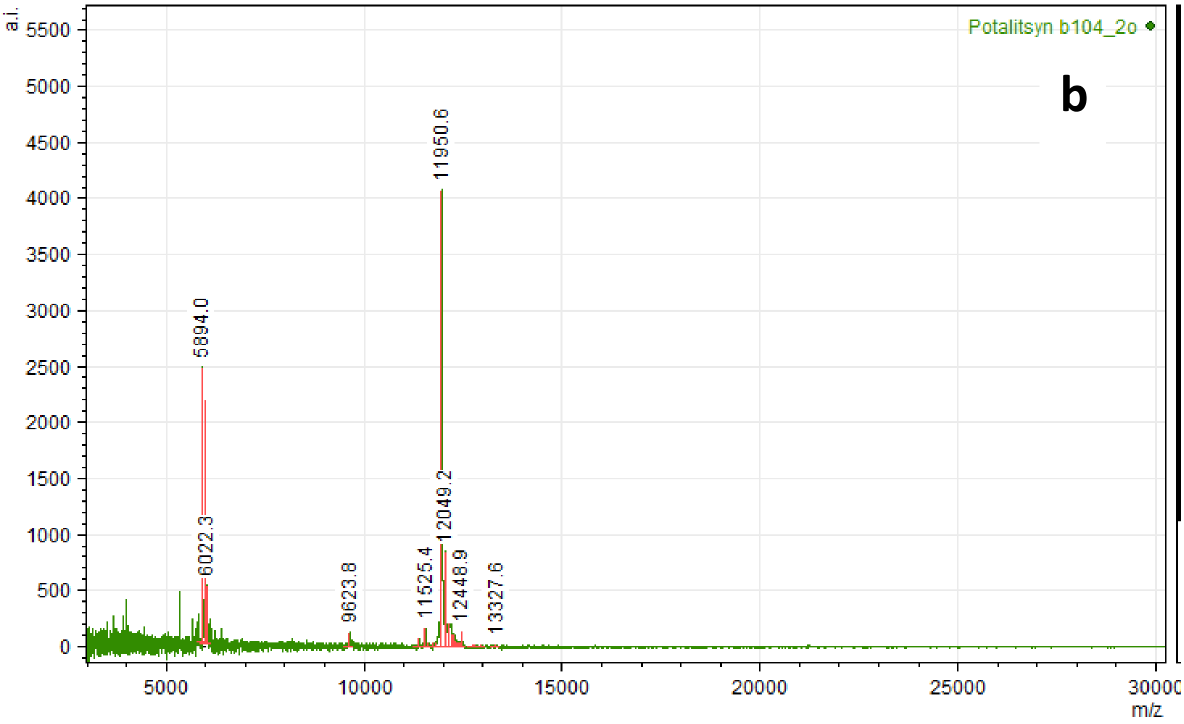

b
a.i.
Potalitsyn b104_2o
5894.0
6022.3
9623.8
11525.4
11950.6
12049.2
12448.9
13327.6
m/z

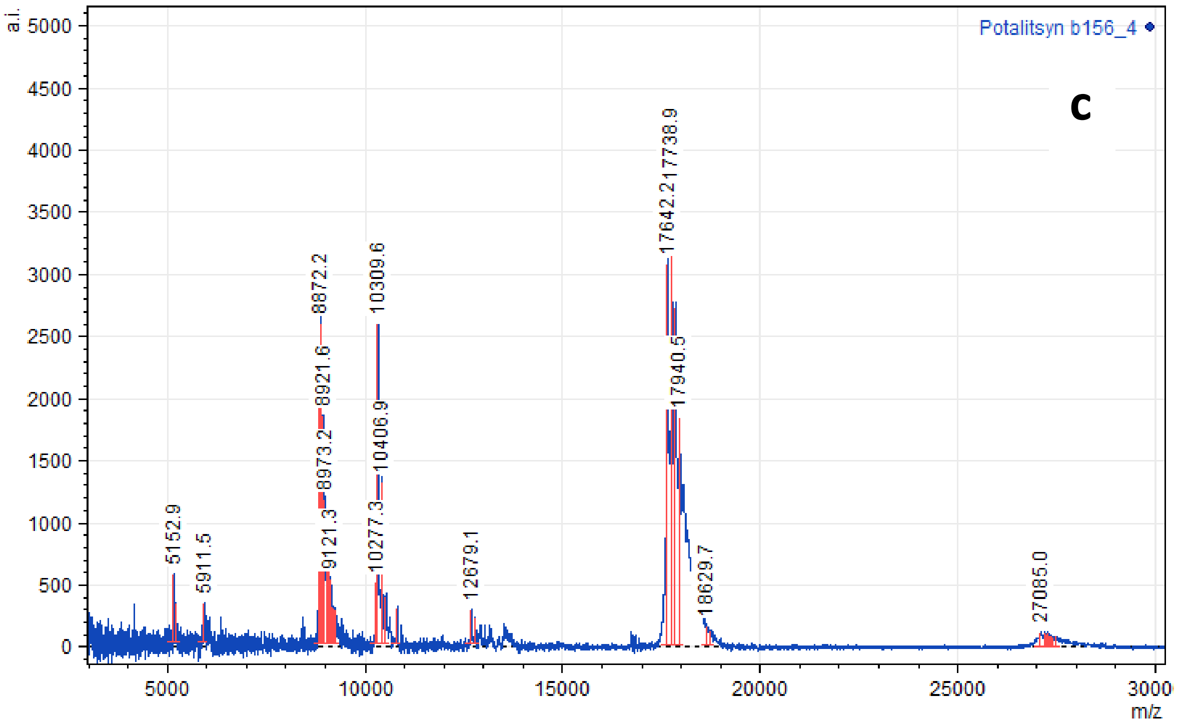

Supplementary Fig. S2 (**a**) Deconvoluted ESI (electrospray ionization) mass spectrum of big-IGF2(87). Expected monoisotopic molecular weight (M) is 9672.7, (**b**) MALDI mass spectrum of big-IGF2(104). Expected average molecular weight (MH^+) is 11950.5, (**c**) MALDI mass spectrum of pro-IGF2(156). Expected average molecular weight (MH^+) is 17635.

Supplementary Note 1. Determination of secondary structures of IGF2 and IGF2 prohormones by Circular Dichroism and theoretical calculations. All spectra were normalized to concentration, optical path length, and the number of amino acids in a particular protein. For IGF2, we can observe a +/-/- pattern with maximum at ~191 nm and minima at ~207 and 223 nm. This corresponds to the presence of a segment with helical conformation in the protein. Nevertheless, the intensity is substantially lower than is typical for highly helical proteins[1]. Therefore, the portion of the α-helical part within the whole IGF2 can be assumed as ~20 %. Big-IGF2(87) and big-IGF2(104) exhibit similar CD patterns to IGF2, only the intensity is slightly lower. Therefore, their α-helical content will obviously be lower. The largest protein in our study, pro-IGF2(156), exhibited a significantly distinct spectrum with zero signal (or only weakly positive) at 185-190 nm and two negative minima at ~203 and ~225 nm. This would suggest another reduction of the α-helical content in the protein and an increase in the contribution of other secondary structures.

Secondary structure content (SSC) was more accurately estimated by decomposing individual CD spectra, using the BeStSel[2, 3] and K2D3 programs[4]. We observed (BeStSel) a gradual decrease of the helical content with the increasing size of the protein, from 25 % for IGF2 to 13 % for pro-IGF2(156). This is partially compensated for by the increasing content of β-strand or "other" conformations. SSC estimated using K2D3 differs from BeStSel. However, K2D3 restricts the analysis to the 190-240 nm region, whereas BeStSel uses a slightly extended region, and therefore we consider the BeStSel estimates to be more reliable. For a detailed analysis, see text and Table S2.

The observed decrease of the helical content for bigger proteins is simply due to an increase in their size with an unchanged proportion of helical content. This means that only parts between Ala1 and Glu67 hold some secondary structure, while the rest is probably unstructured. This was confirmed by calculating the minimal helical content hc (see Methods and Table S3 for details) that corresponds relatively well to α-helix SSC obtained with the BeStSel analysis. We also tried to predict 3D structures of big-IGF2s and pro-IGF2(156) (and IGF2 as a control) using ColabFold[5], which combines the homology search of MMseq2[6] with AlphaFold2[7]. Supplementary Fig. S3a shows an overlay of the predicted IGF2 structure with the reported NMR structure (2L29), revealing their high degree of resemblance that is confirmed by the analysis of the structures (Supplementary Fig. S4). Calculated hc for

predicted IGF2 corresponds well to that estimated by CD (or to the minimal hc; see Table S3). Analysis of the predicted structure of big-IGF2(87) (cf. Supplementary Figs. S4b and S5) and its calculated hc of 26 % suggest that it also corresponds well to the actual protein structure, even though the accuracy rate decreases starting from a position around ~60. Only the parts of big-IGF2(104) and pro-IGF2(156) corresponding to IGF2 (Ala1 - Glu67) were reliably predicted (Supplementary Figs. S5a-f), including the relative positions of the helical segments (Supplementary Figs. S5g-i). Models predicted additional helical segments for big-IGF2(104) (Phe90-Leu102) and pro-IGF2(156) (Tyr92-Arg130), resulting in unrealisticaly high hc of 33 % and 38 %, respectively. To conclude, the secondary structure between Ala1 and Glu67 in big-IGF2s and pro-IGF2 are largely similar to IGF2, whereas their remaining parts are rather unstructured.

To interpret the CD spectra of our proteins in greater depth, we estimated the secondary structure content (SSC), using the BeStSel[2] and K2D3[8] programs. Both programs approximate the experimental CD spectra by a linear combination of a set of basic spectra, corresponding to proteins with a solved structure and thus with a known secondary structure composition. BeStSel uses 8 secondary structure motifs (2 α-helices, 3 antiparallel and 1 parallel β-strands, 1 turn, and others; according to[4]. The K2D3 program only distinguishes α-helical, β-stranded, and “other” conformations. Note that neither method is able to recognize other helical conformations (e.g. PPII, 3_{10}) and these are gathered as “others”. Table S1 summarizes the estimated SSC for all proteins and both methods. We can see a gradual decrease of the helical content with increasing size of the protein. This is compensated for by the increasing content of β-strand or others (depending on the method). As K2D3 limits the analysis to the region of 190-240 nm, while BeStSel uses an extended region of 185-250 nm, we find the BeStSel estimations more reliable. For brevity, we have grouped both α-helices into one family of conformation, as well as all β-strands. Fig. 2 presents these grouped SSC estimates.

The observed decrease of the helical content for bigger proteins is simply due to an increase in their size, with an unchanged proportion of helical content. This means that only parts between ALA1 and GLU67 hold some secondary structure, while the rest is probably unstructured (or contains secondary structures labeled as “others”). We calculated the minimal helical content hc (see Table S2), based precisely on the assumption that the proportion of the helix is unchanged, and only the size of the protein increases. We obtained

hc of 27-36 % (IGF2), 21-28 % (big-IGF2(87)), 17-23 % (big-IGF2(104)), and 12-15 % (pro-IGF2(156)). Specific hc (and its range) depends on the reference structure used. Obtained hc corresponds relatively well to α-helix SSC obtained with BeStSel, which supports the idea of an unstructured "tail" of big-IGFs.

Supplementary Table S1. Secondary structure content (in %) estimated for all studied proteins according to experimental CD.

			BeStSel		
**185-250 nm**		**IGF2**	**big-IGF2(87)**	**big-IGF2(104)**	**pro-IGF2(156)**
α-helix	regular	14	12	11	5
	distorted	11	11	10	8
	sum	25	23	21	13
β-strand	antiparallel	18	18	18	23
	parallel	0	0	0	0
	sum	18	18	18	23
turn		15	14	14	17
others		42	45	47	48
			**K2D3**		
**190-240 nm**					
α-helix		18	14	12	3
β-strand		21	21	24	32
others		61	65	64	65

Alpha fold predictions

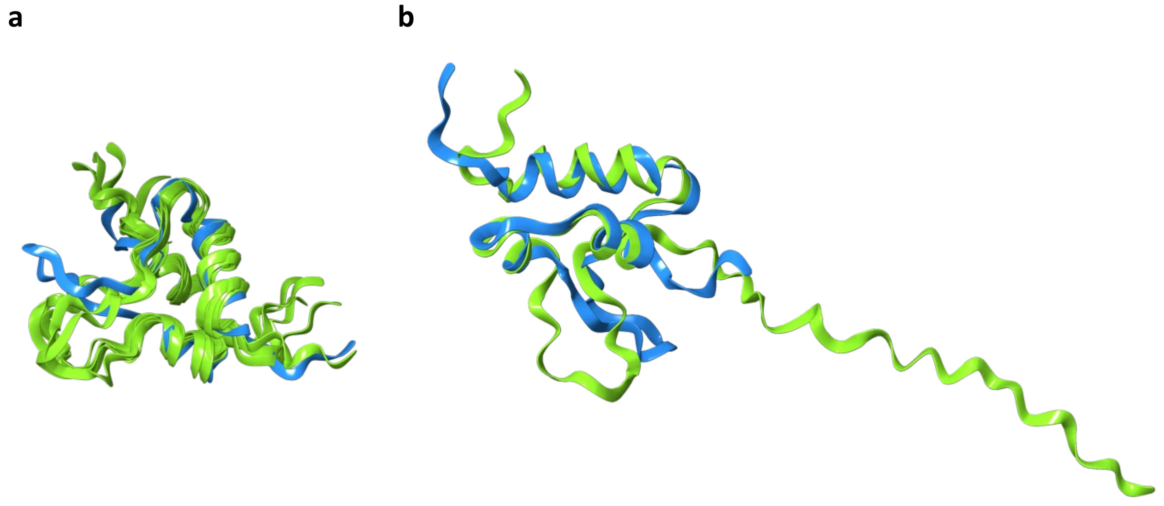

Supplementary Fig. S3 Comparison of IGF2 NMR structure (2L29; blue structures) with predicted (AlphaFold2; green) structure of (**a**) five predicted structures of IGF2 and (**b**) big-IGF2(87).

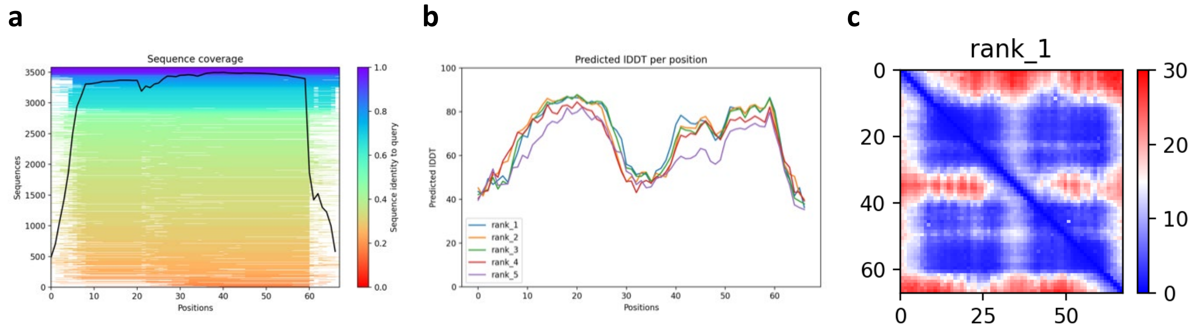

Supplementary Fig. S4 Analysis of predicted (AlphaFold2) IGF2 structure used as a control: the sequence coverage diagram (**a**), predicted LDDT curves for five IGF2 structures (**b**), predicted aligned error for the highest ranked IGF2 structure (**c**).

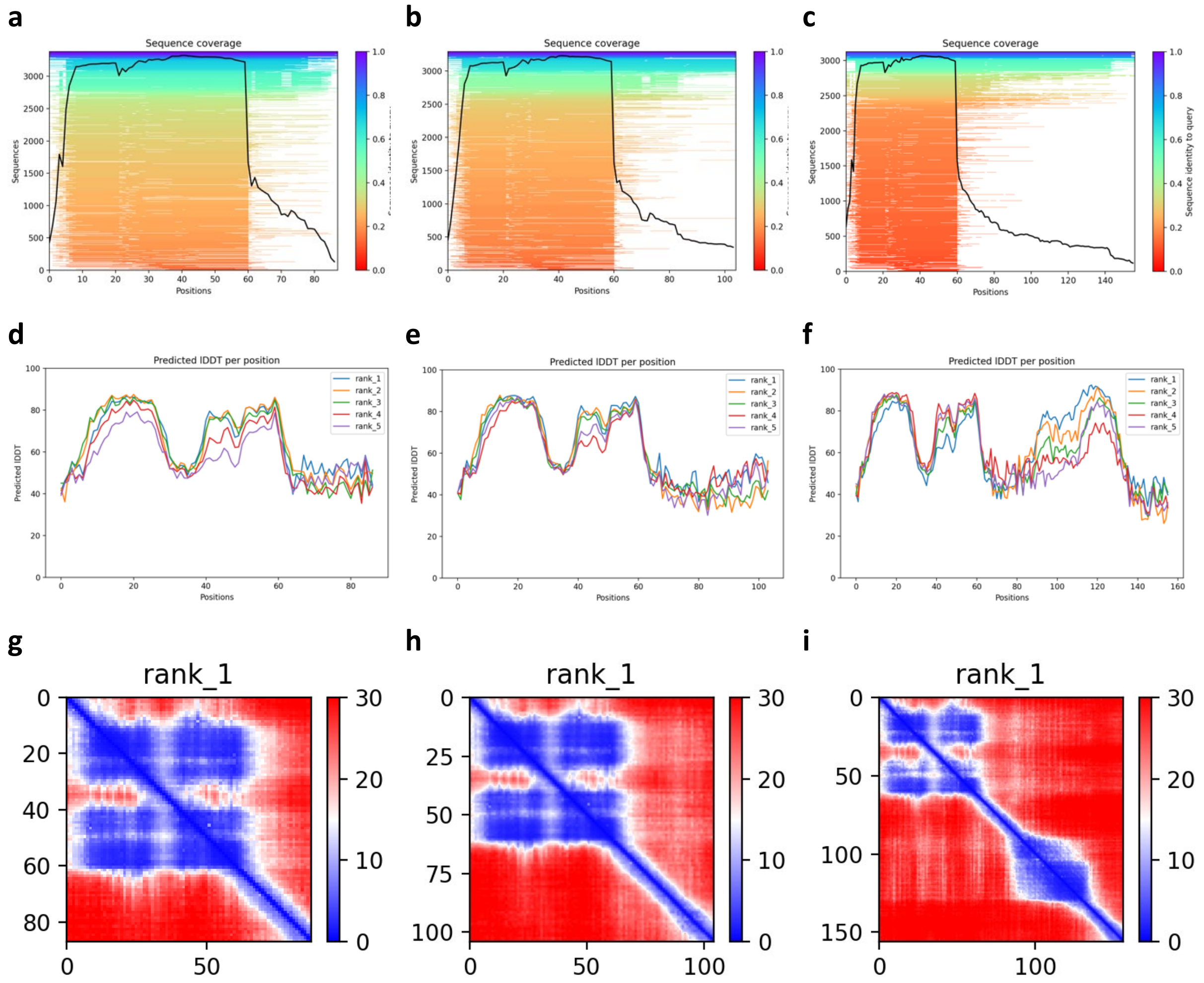

Supplementary Fig. S5 Analysis of structures predicted using AlphaFold2. Top row: the sequence coverage diagram of (**a**) big-IGF2(87), (**b**) big-IGF2(104), and (**c**) pro-IGF2(156); Middle row: predicted LDDT curves for five structures of (**d**) big-IGF2(87), (**e**) big-IGF2(104), and (**f**) pro-IGF2(156); Bottom row: predicted aligned error for the highest ranked structure of (**g**) big-IGF2(87), (**h**) big-IGF2(104), and (**i**) pro-IGF2(156).

Supplementary Table S2. Secondary structure content (in %) estimated for all studied proteins using different methods

	helical content (%)			
**Model**	**IGF2**	**big-IGF2(87)**	**big-IGF2(104)**	**pro-IGF2(156)**
CD (BeStSel)	25	23	21	13
Minimal *hc*	27-36	21-28	17-23	12-15
AlphaFold2	33	26	33	38

[a]*hc* = number of amino acids in α-helical conformation as in IGF2 (pdb structures - 2L29, 2V5P, 6VWG) / number of all amino acids (67, 87, 104, and 156). The wider range of values is due to the unequal number of "helical" amino acids in different pdb structures.

Supplementary Table S3. Receptor-binding affinities of IGF1, IGF2, human insulin, big-IGF2(87), big-IGF2(104), and pro-IGF2(156) on individual receptors or binding proteins. The K_d values of individual proteins were determined in (n) independent series of measurements. The affinity of the native hormones for their cognate receptors was set at 100 % and relative binding affinities of ligands were calculated and are shown in the main text (Table 1). gly means glycosylated.

	IR-A K_d ± S.D. (*n*)	IR-B K_d ± S.D. (*n*)	IGF1R K_d ± S.D. (*n*)	IGFBP3 K_d ± S.D. (*n*)	D11:IGF2R K_d ± S.D. (*n*)	M6P/IGF2R K_d ± S.D. (*n*)	IGFBP3:ALS K_d ± S.D. (*n*)
IGF2	3.17 ± 1.25 (3)	7.02 ± 2.93 (3)	0.76 ± 0.12[a] (3)	0.2 ± 0.10 (4) (100%)	1.29 ± 0.27 (7) (100%)	0.94 ± 0.36 (4) (100%)	0.95 ± 0.31 (3) (100%)
IGF1	23.8 ± 6.6 (3)*	224 ± 16 (4)*	0.12 ± 0.02[a] (3) (100%)<br>0.24 ± 0.08[b] (5) (100%)	0.31 ± 0.07 (3)	n.b.	31.4 ± 2.61 (3)	n.d.
HI	0.27 ± 0.06 (4) (100%)	0.26 ± 0.07 (4) (100%)	292 ± 31[b] (3)*	n.d.	n.d.	n.d.	n.d.
big-IGF2(87)	3.82 ± 0.23 (3)	9.47 ± 3.30 (3)	1.60 ± 0.55[b] (5)	0.29 ± 0.17 (3)	1.77 ± 0.74 (4)	0.56 ± 0.25 (5)	1.63 ± 0.99 (3)
big-IGF2(104)	2.75 ± 0.28 (3)	8.83 ± 2.74 (3)	2.48 ± 0.32[b] (6)	0.14 ± 0.05 (3)	0.19 ± 0.07 (4)	0.14 ± 0.03 (6)	5.58 ± 0.99 (3)
pro-IGF2(156)	12.21 ± 1.93 (3)	16.04 ± 3.97 (2)	67.9 ± 27.2[b] (5)	1.25 ± 0.63 (3)	2.31 ± 1.68 (3)	6.33 ± 4.71 (4)	10.5 ± 3.14 (3)
gly pro-IGF2(156)	25 (1)	n.d.	111 (1)	1.55 (1)	n.d.	50% inhibition at 10^{-6} M	n.d.

The K_d values and were calculated from at least three independent measurements (n, number of replicates). [a, b] The individual K_d values of ligands in this column were determined in two independent series of experiments and are relative to a corresponding native IGF1 K_d value. ([a] to [a] and [b] to [b]). n.b. means no binding. n.d. means not determined. *Data from Ref.[9].

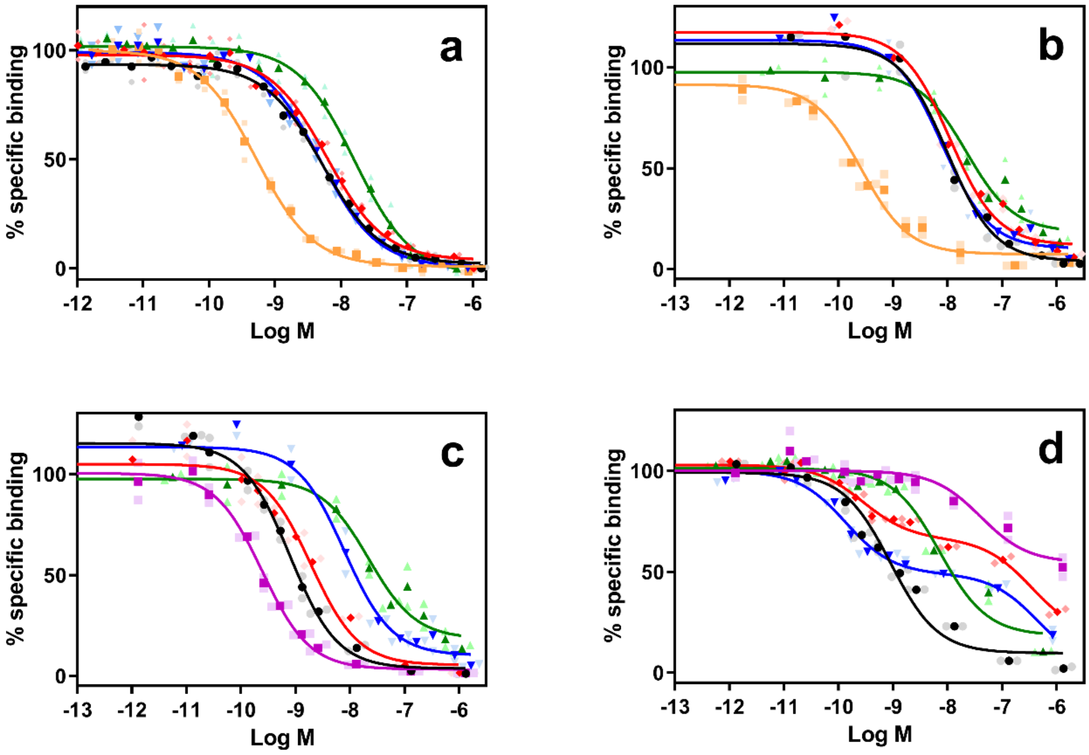

Supplementary Fig. S6 Binding curves of IGF2 forms for (**a**) IR-A, (**b**) IR-B, (**c**) IGF1-R, (**d**) M6P/IGF2R. Inhibition of binding of human [^{125}I]-monoiodotyrosyl-ligand to corresponding receptor by human insulin (orange), IGF1 (magenta), IGF2 (black), big-IGF2(87) (red), big-IGF2(104) (blue), pro-IGF2(156) (green). Representative binding curve for each hormone or analog is shown, n ≥ 3 independent experiments with 2 replicates.

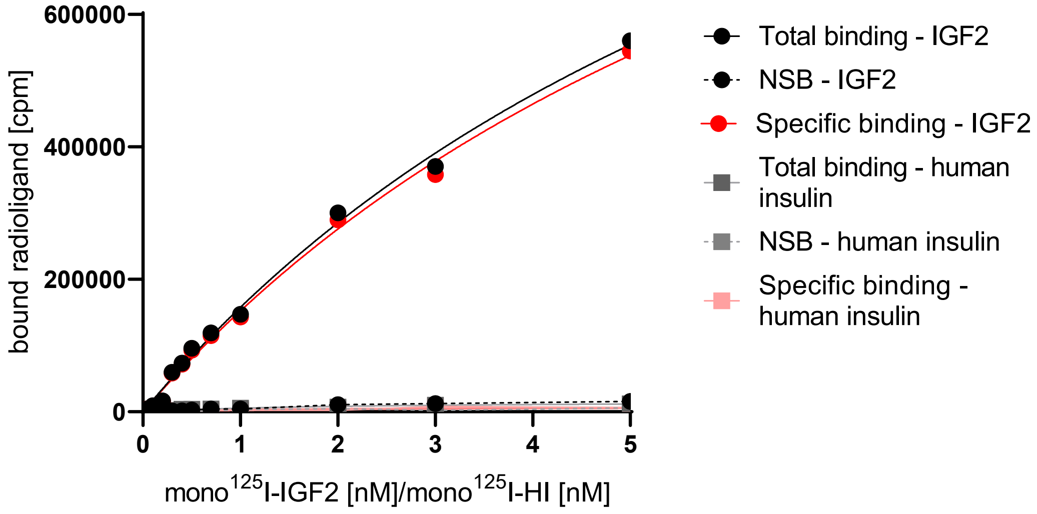

Supplementary Fig. S7 A typical saturation binding curve of [^{125}I]-monoiodotyrosyl-Tyr2-IGF2 and [^{125}I]-monoiodotyrosyl-TyrA14-HI to R-cells. Statistical analysis of the results from three such independent experiments in monoplicates provided the final K_d value 8.20 ± 0.85 nM (n = 3) for IGF2 to IGF2R.

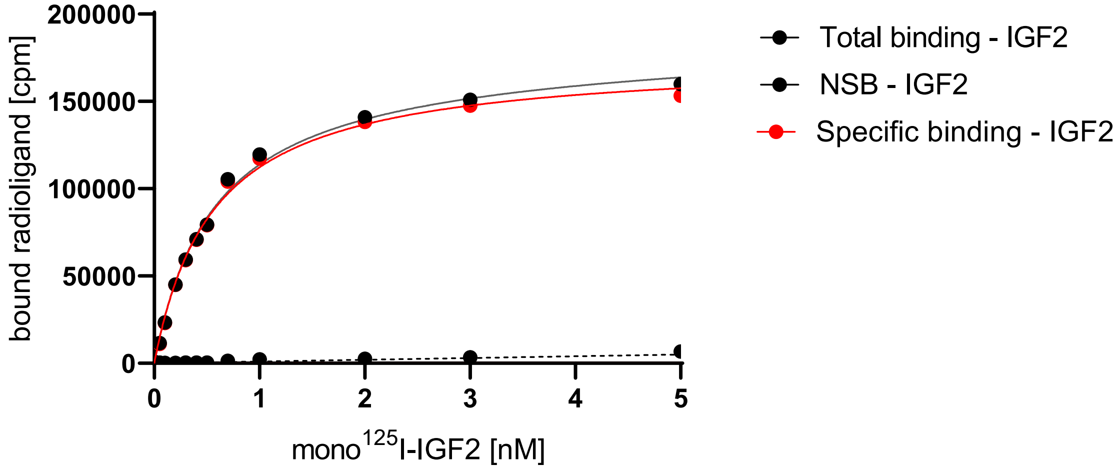

Supplementary Fig. S8 A typical saturation binding curve of [^{125}I]-monoiodotyrosyl-Tyr2-IGF2 to IGFBP3. Statistical analysis of the results from three independent experiments in monoplicates provided the final K_d value 0.59 ± 0.29 nM (n = 3) for IGF2 to IGFBP3.

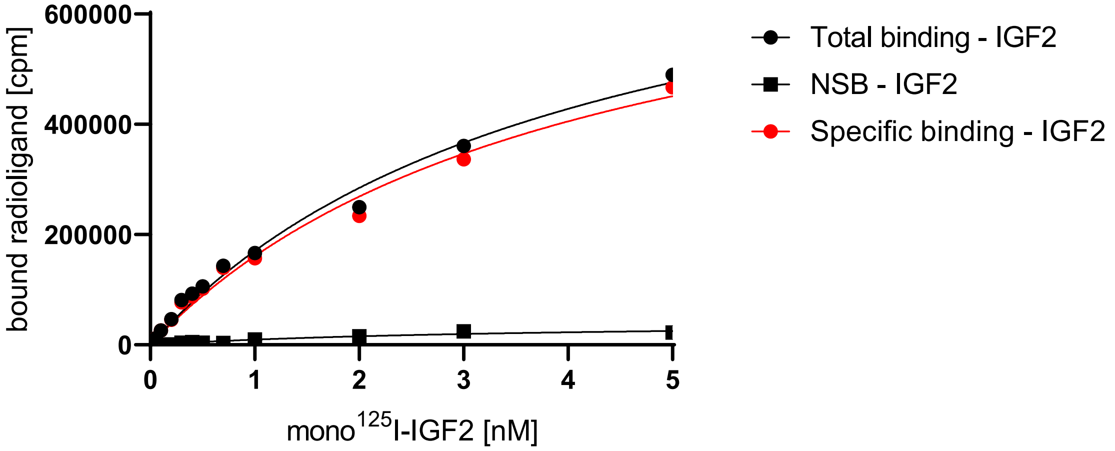

Supplementary Fig. S9 A typical saturation binding curve of [^{125}I]-monoiodotyrosyl-Tyr2-IGF2 with IGFBP3 to ALS. Two independent measurements in monoplicates gave us the mean K_d value. K_d value was set at value 5.90 ± 2.53 nM (n = 2) for IGF2:IGFPB3 to ALS.

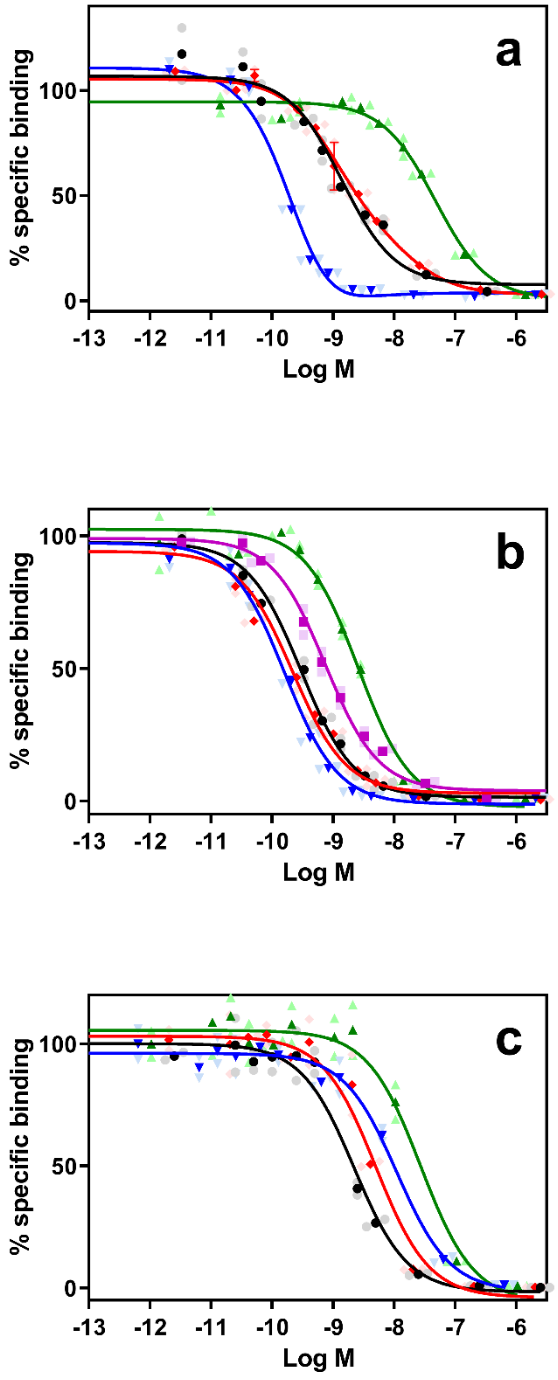

Supplementary Fig. S10 Binding curves for (**a**) D11:IGF2R, (**b**) IGFBP3, (**c**) ALS:IGFBP3. Inhibition of binding of human [^{125}I]-monoiodotyrosyl-ligand to corresponding receptor by IGF1 (magenta), IGF2 (black), big-IGF2(87) (red), big-IGF2(104) (blue), pro-IGF2(156) (green). Representative binding curve for each hormone or analog is shown, n ≥ 3 independent experiments with 2 replicates.

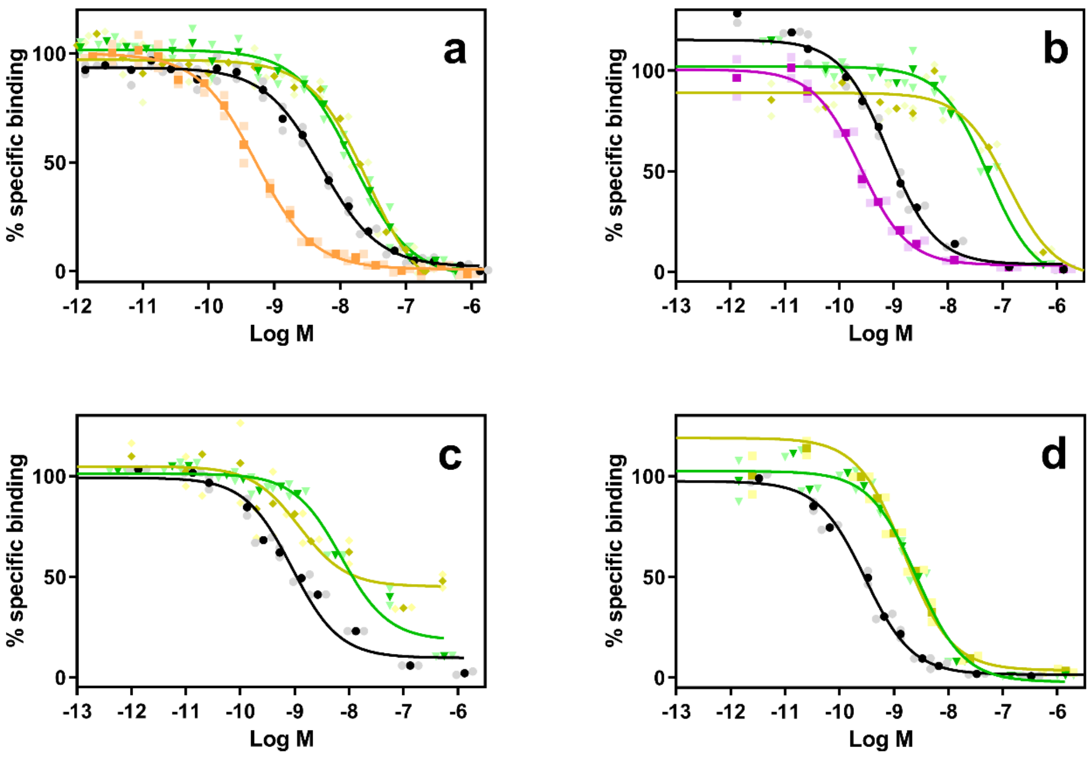

Supplementary Fig. S11 Binding curves for (**a**) IR-A, (**b**) IGF1R, (**c**) M6P/IGF2R and (**d**) IGFBP3. Inhibition of binding of human [^{125}I]-monoiodotyrosyl-ligand to corresponding receptor by human insulin (orange), IGF1 (magenta), IGF2 (black), nongly pro-IGF2(156) (green), gly pro-IGF2(156) (dark yellow). Representative binding curve for each hormone or analog is shown, n ≥ 3 independent experiments with 2 replicates.

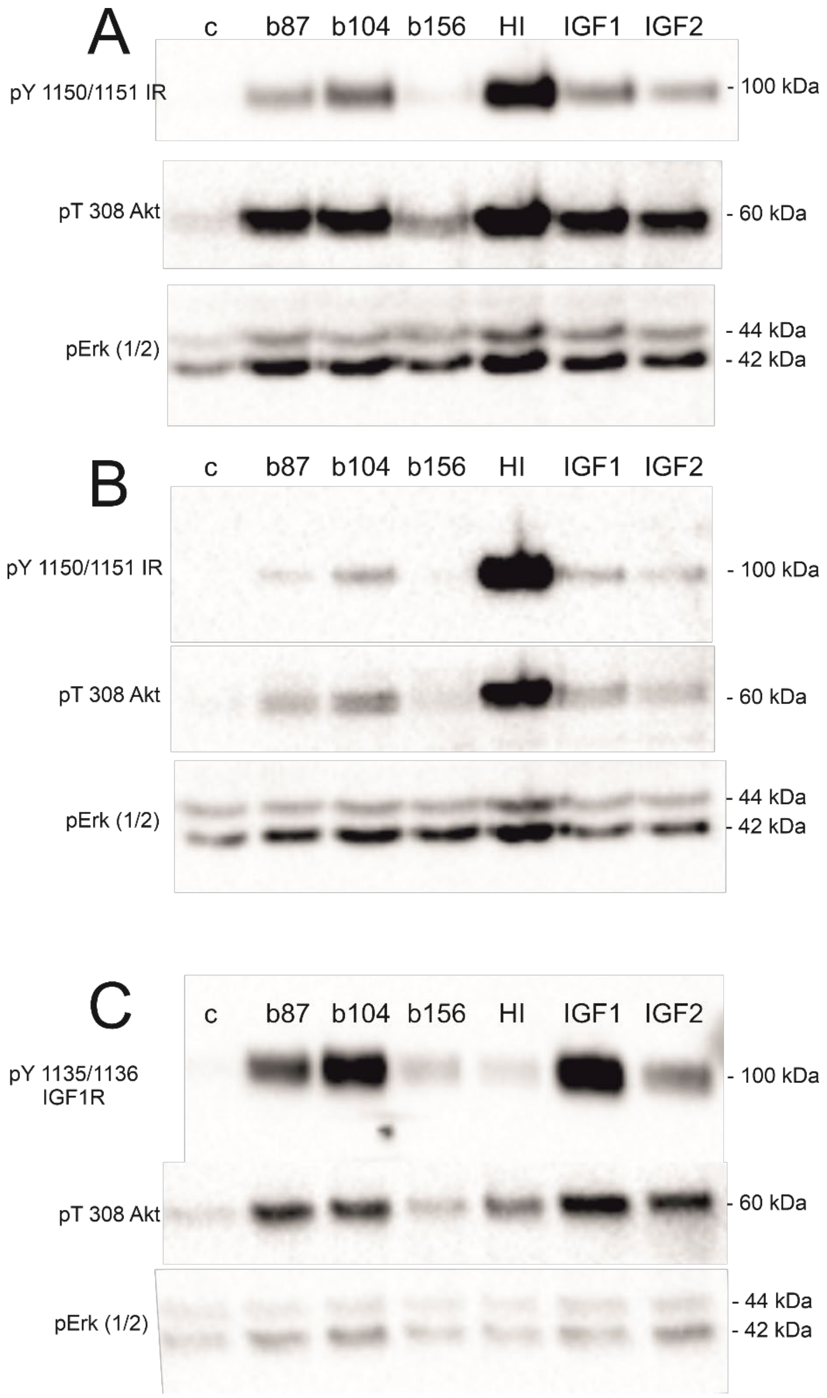

Supplementary Fig. S12 Representative Western blots for relative abilities of IGF2 proforms to stimulate receptors' phosphorylation; **(A)** IR-A transfected fibroblasts; **(B)** IR-B transfected fibroblasts; **(C)** IGF1R transfected fibroblasts. Cells were stimulated with 10 nM ligands for 10 min; **(D)** Uncropped and unedited blots. All western blots showing receptor autophosphorylation induced by wild-type hormones and IGF2 proforms in fibroblasts transfected with individual human receptors IR-A, IR-B and IGF-1R. Membranes were cut at 75 kDa and upper parts were incubated with the anti phospho-receptor antibody. Membranes were recorded together as shown in the figure. For analysis the images were cropped and evaluated individually. Each batch originated from different passage of cells; **(E)** All western blots showing activation of Akt induced by wild-type hormones and IGF2 proforms in fibroblasts

transfected with individual human receptors IR-A, IR-B and IGF-1R. Membranes were cut bettwen 75 kDa and 50 kDa and incubated with the anti phospho-Akt antibody. Membranes were recorded together as shown in the figure. For analysis the images were cropped and evaluated individually. Each batch originated from different passage of cells; **(F)** All western blots showing activation of Erk induced by wild-type hormones and IGF2 proforms in fibroblasts transfected with individual human receptors IR-A, IR-B and IGF-1R. Membranes were cut at 50 kDa and lower parts were incubated with the anti phospho-Erk antibody. Membranes were recorded together as shown in the figure. For analysis the images were cropped and evaluated individually. Each batch originated from different passage of cells.

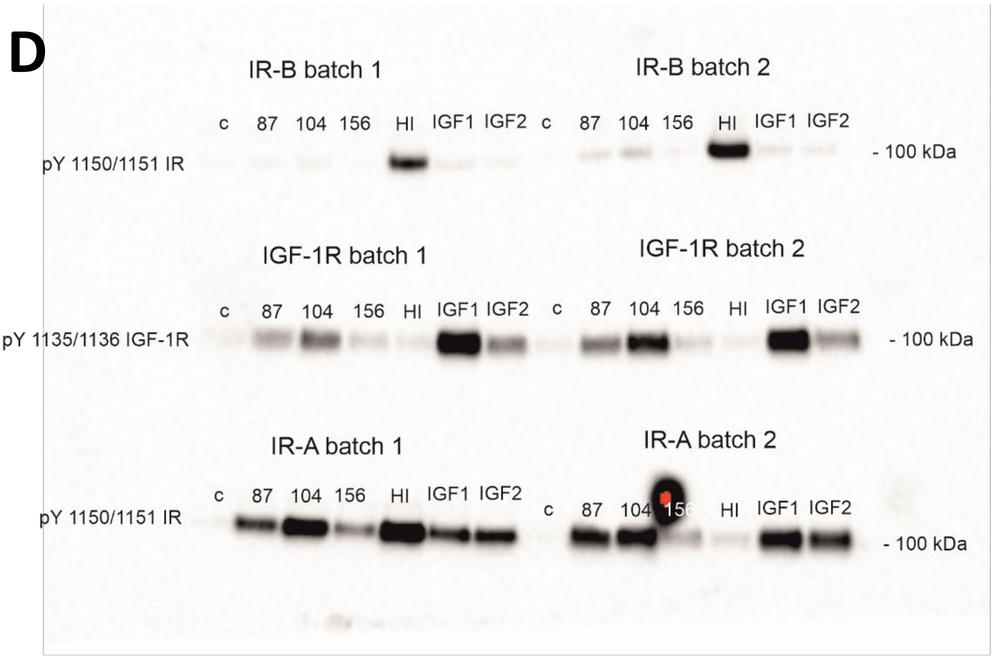
D
IR-B batch 1
IR-B batch 2
c 87 104 156 HI IGF1 IGF2 c 87 104 156 HI IGF1 IGF2
pY 1150/1151 IR
- 100 kDa
IGF-1R batch 1
IGF-1R batch 2
c 87 104 156 HI IGF1 IGF2 c 87 104 156 HI IGF1 IGF2
pY 1135/1136 IGF-1R
- 100 kDa
IR-A batch 1
IR-A batch 2
c 87 104 156 HI IGF1 IGF2 c 87 104 156 HI IGF1 IGF2
pY 1150/1151 IR
- 100 kDa

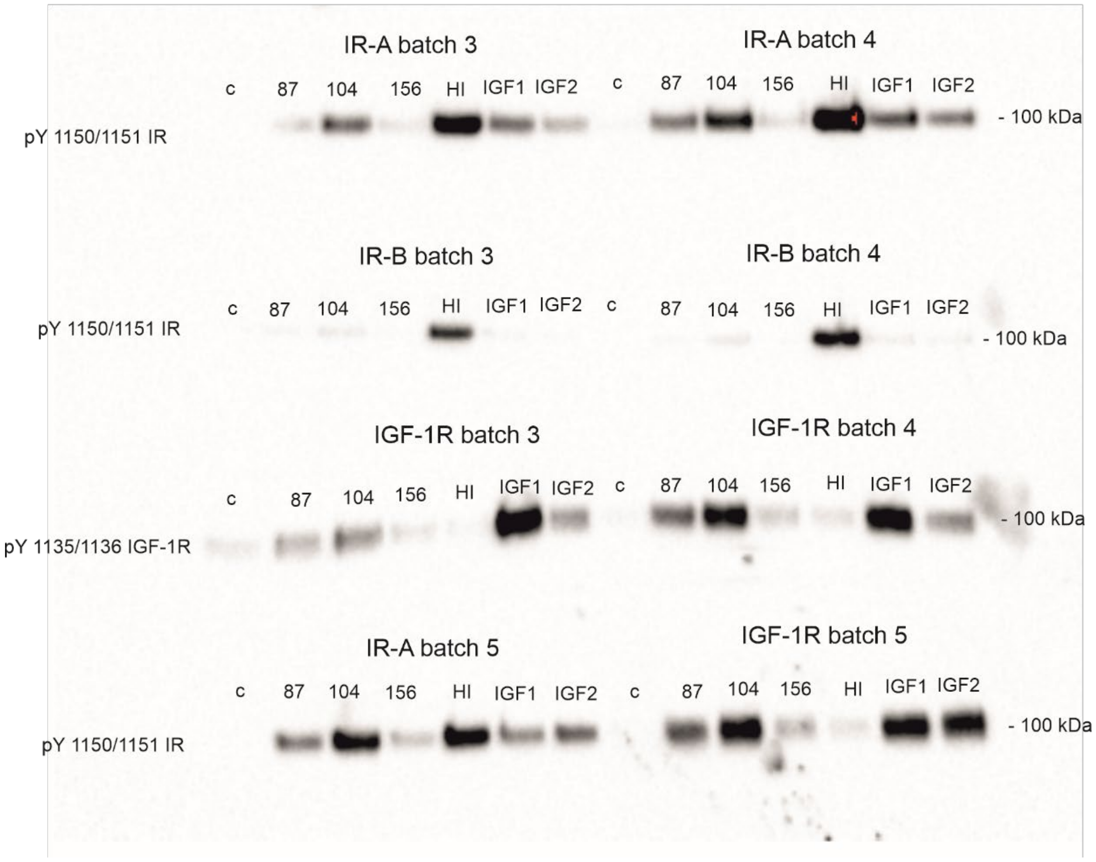
IR-A batch 3
IR-A batch 4
c 87 104 156 HI IGF1 IGF2 c 87 104 156 HI IGF1 IGF2
pY 1150/1151 IR
- 100 kDa
IR-B batch 3
IR-B batch 4
c 87 104 156 HI IGF1 IGF2 c 87 104 156 HI IGF1 IGF2
pY 1150/1151 IR
- 100 kDa
IGF-1R batch 3
IGF-1R batch 4
c 87 104 156 HI IGF1 IGF2 c 87 104 156 HI IGF1 IGF2
pY 1135/1136 IGF-1R
- 100 kDa
IR-A batch 5
IGF-1R batch 5
c 87 104 156 HI IGF1 IGF2 c 87 104 156 HI IGF1 IGF2
pY 1150/1151 IR
- 100 kDa

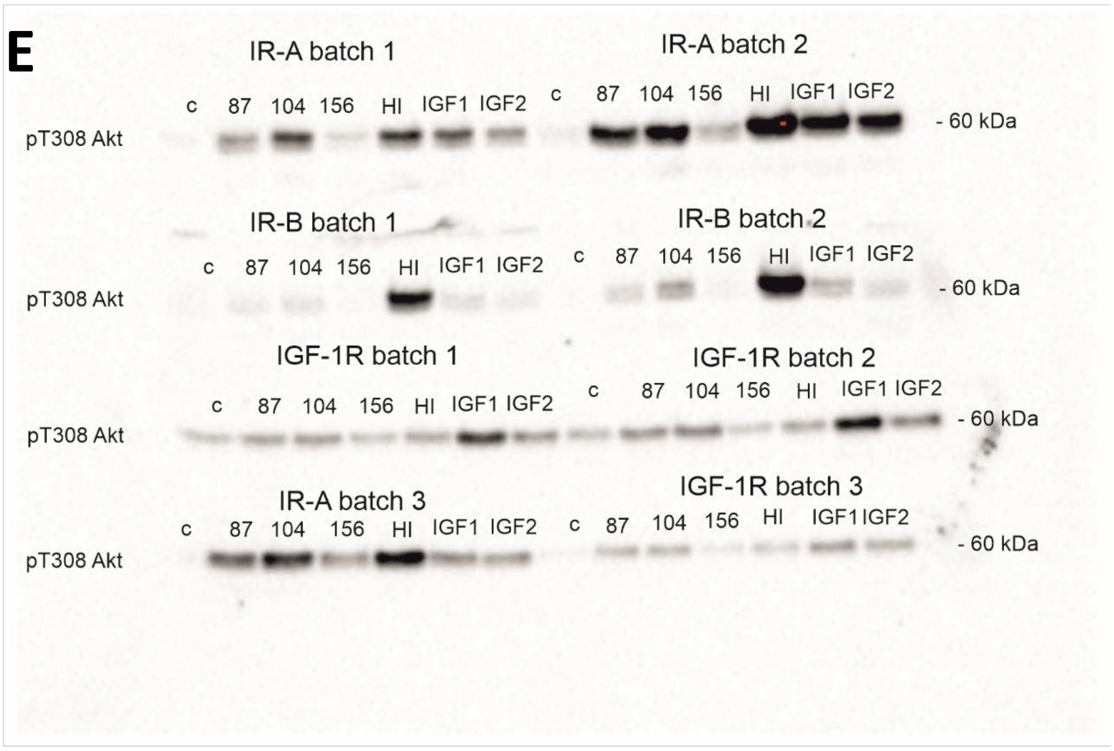
E
IR-A batch 1
IR-A batch 2
c 87 104 156 HI IGF1 IGF2 c 87 104 156 HI IGF1 IGF2
pT308 Akt
- 60 kDa
IR-B batch 1
IR-B batch 2
c 87 104 156 HI IGF1 IGF2 c 87 104 156 HI IGF1 IGF2
pT308 Akt
- 60 kDa
IGF-1R batch 1
IGF-1R batch 2
c 87 104 156 HI IGF1 IGF2 c 87 104 156 HI IGF1 IGF2
pT308 Akt
- 60 kDa
IR-A batch 3
IGF-1R batch 3
c 87 104 156 HI IGF1 IGF2 c 87 104 156 HI IGF1 IGF2
pT308 Akt
- 60 kDa

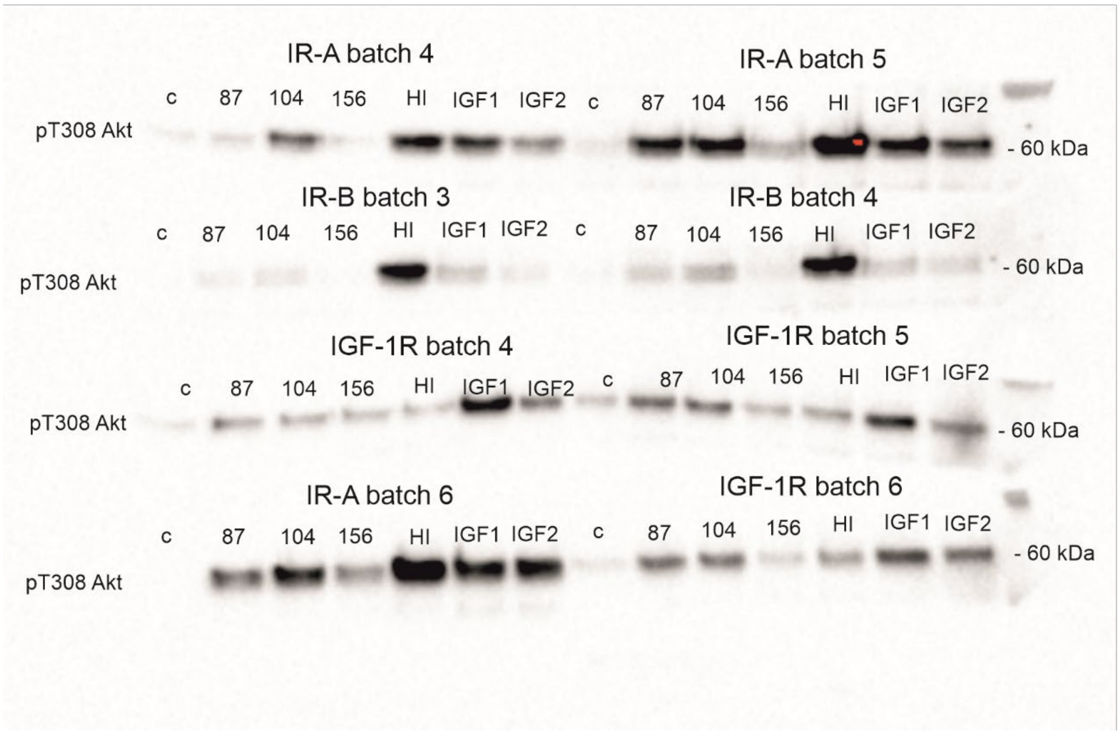
IR-A batch 4
IR-A batch 5
c 87 104 156 HI IGF1 IGF2 c 87 104 156 HI IGF1 IGF2
pT308 Akt
- 60 kDa
IR-B batch 3
IR-B batch 4
c 87 104 156 HI IGF1 IGF2 c 87 104 156 HI IGF1 IGF2
pT308 Akt
- 60 kDa
IGF-1R batch 4
IGF-1R batch 5
c 87 104 156 HI IGF1 IGF2 c 87 104 156 HI IGF1 IGF2
pT308 Akt
- 60 kDa
IR-A batch 6
IGF-1R batch 6
c 87 104 156 HI IGF1 IGF2 c 87 104 156 HI IGF1 IGF2
pT308 Akt
- 60 kDa

F

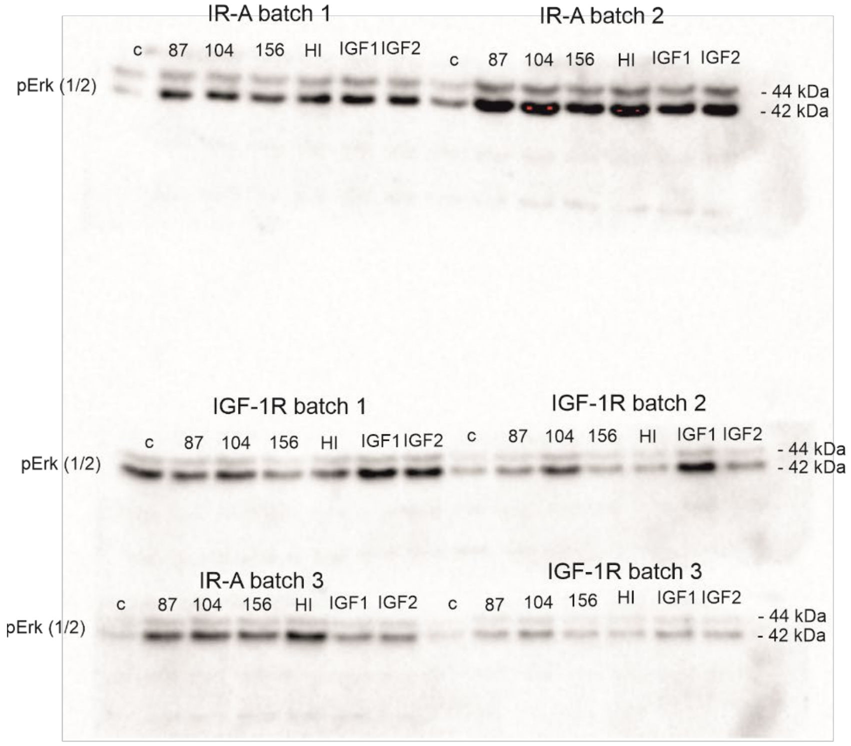
IR-A batch 1
IR-A batch 2
c 87 104 156 HI IGF1 IGF2 c 87 104 156 HI IGF1 IGF2
pErk (1/2)
- 44 kDa
- 42 kDa
IGF-1R batch 1
IGF-1R batch 2
c 87 104 156 HI IGF1 IGF2 c 87 104 156 HI IGF1 IGF2
pErk (1/2)
- 44 kDa
- 42 kDa
IR-A batch 3
IGF-1R batch 3
c 87 104 156 HI IGF1 IGF2 c 87 104 156 HI IGF1 IGF2
pErk (1/2)
- 44 kDa
- 42 kDa

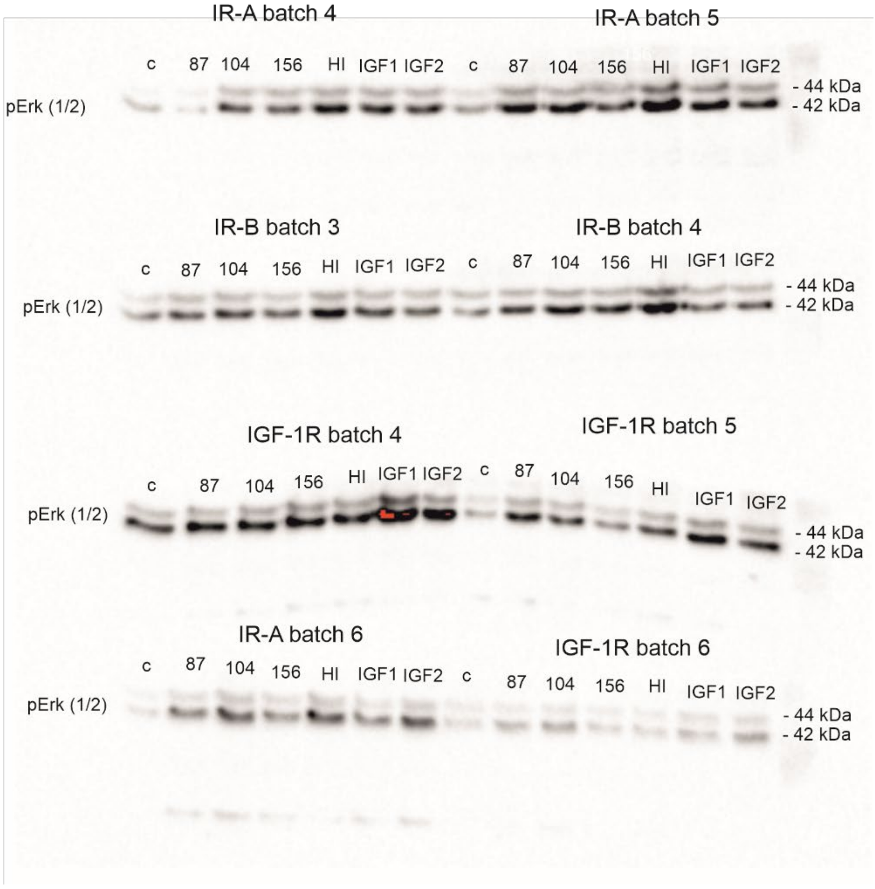
IR-A batch 4
IR-A batch 5
c 87 104 156 HI IGF1 IGF2 c 87 104 156 HI IGF1 IGF2
pErk (1/2)
- 44 kDa
- 42 kDa
IR-B batch 3
IR-B batch 4
c 87 104 156 HI IGF1 IGF2 c 87 104 156 HI IGF1 IGF2
pErk (1/2)
- 44 kDa
- 42 kDa
IGF-1R batch 4
IGF-1R batch 5
c 87 104 156 HI IGF1 IGF2 c 87 104 156 HI IGF1 IGF2
pErk (1/2)
- 44 kDa
- 42 kDa
IR-A batch 6
IGF-1R batch 6
c 87 104 156 HI IGF1 IGF2 c 87 104 156 HI IGF1 IGF2
pErk (1/2)
- 44 kDa
- 42 kDa

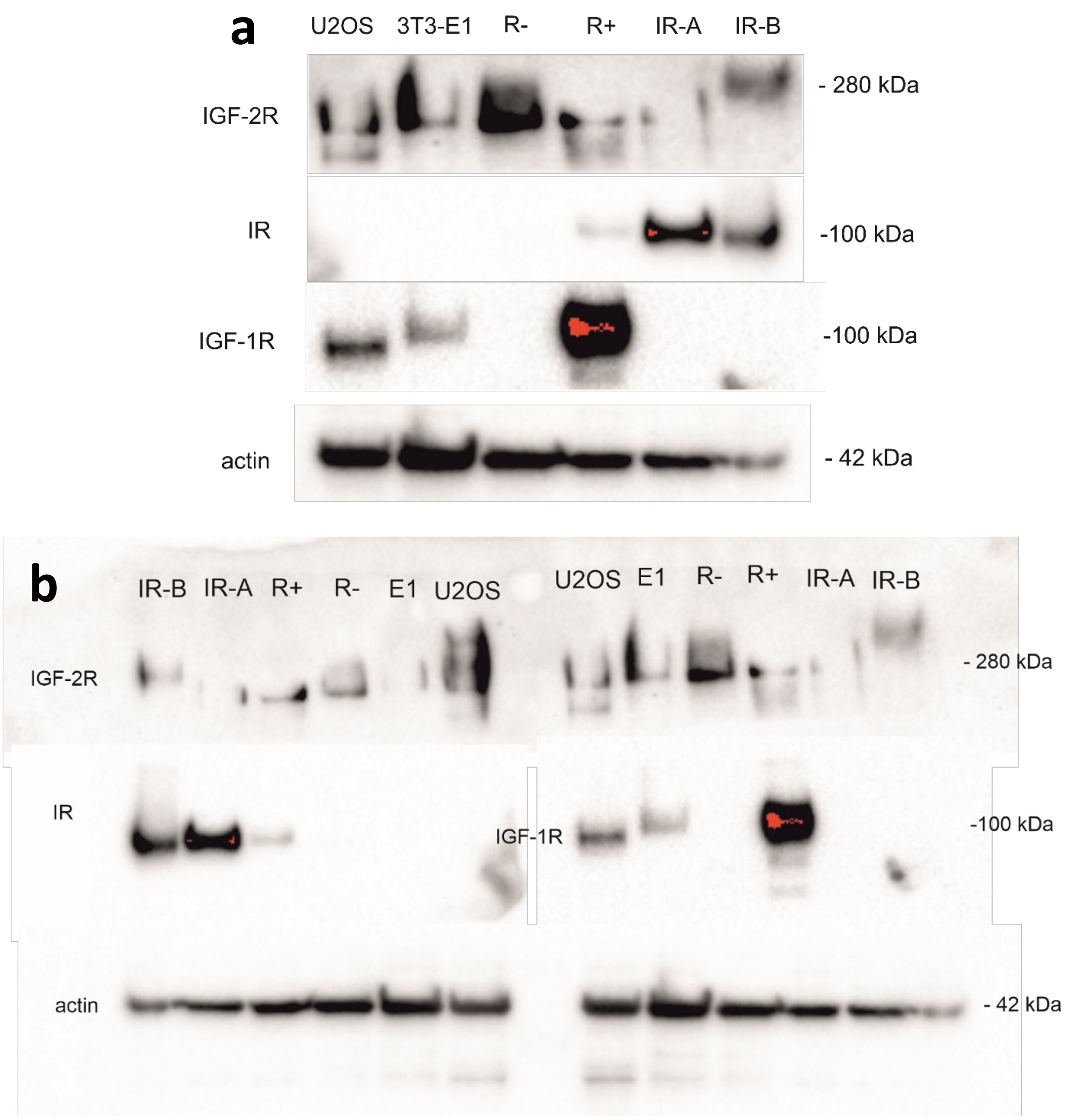

Supplementary Fig. S13 (a) Representative Western blot showing expression of particular receptors in different cells. Cells were stimulated with 10 nM ligands for 10 min; **(b)** Uncropped and unedited blot. The membrane was cut at the 150 kDa, 50 kDa and 25 kDa markers. Upper part was incubated with the IGF-2R antibody (Cell Signaling Technology #14364), the part between the 150 and 50 kDa markers was cut vertically in half and incubated with the IR (Cell Signalling Technology #3025) respective IGF-1R (Cell Signaling Technology #3018) antibodies and the part bellow 50 kDa was incubated with anti-actin antibody (Sigma, #A5060).

Supplementary References

1. Greenfield NJ. Using circular dichroism spectra to estimate protein secondary structure. *Nat. Protoc.* **1**, 2876-2890 (2006).

2. Micsonai A*, et al.* BeStSele: webserver for secondary structure and fold prediction for protein CD spectroscopy. *Nucleic Acids Res.* **50**, W90-W98 (2022).

3. Micsonai A*, et al.* BeStSel: a web server for accurate protein secondary structure prediction and fold recognition from the circular dichroism spectra. *Nucleic Acids Res.* **46**, W315-W322 (2018).

4. Kabsch W, Sander C. Dictionary of Protein Secondary Structure - Pattern-Recognition of Hydrogen-Bonded and Geometrical Features. *Biopolymers* **22**, 2577-2637 (1983).

5. Mirdita M, Schutze K, Moriwaki Y, Heo L, Ovchinnikov S, Steinegger M. ColabFold: making protein folding accessible to all. *Nat. Met.* **19**, 679-682 (2022).

6. Mirdita M, Steinegger M, Soding J. MMseqs2 desktop and local web server app for fast, interactive sequence searches. *Bioinformatics* **35**, 2856-2858 (2019).

7. Jumper J*, et al.* Highly accurate protein structure prediction with AlphaFold. *Nature* **596**, 583-589 (2021).

8. Louis-Jeune C, Andrade-Navarro MA, Perez-Iratxeta C. Prediction of protein secondary structure from circular dichroism using theoretically derived spectra (vol 80, pg 374, 2012). *Proteins-Structure Function and Bioinformatics* **80**, 2818-2818 (2012).

9. Krizkova K*, et al.* Insulin-Insulin-like Growth Factors Hybrids as Molecular Probes of Hormone:Receptor Binding Specificity. *Biochemistry* **55**, 2903-2913 (2016).
